# The Role of Steroidogenic Expression, Apoptotic, and Inflammatory Mediators in Polyphenol-Rich Extract of *Ocimum gratissimum* Mitigation of Cadmium-Induced Reprotoxicity in Male Rats

**DOI:** 10.1155/vmi/9165137

**Published:** 2025-06-25

**Authors:** Ikokide Emmanuel Joseph, Jaja Ishmael Festus, Temitayo Olabisi Ajibade, Ademola Adetokunbo Oyagbemi, Emmanuel Egwu Ewa, Mathew Olugbenga Oyeyemi

**Affiliations:** ^1^Department of Theriogenology, Faculty of Veterinary Medicine, University of Ibadan, Ibadan, Oyo, Nigeria; ^2^Department of Livestock and Pasture Science, University of Fort Hare, Alice 5700, South Africa; ^3^Department of Agriculture and Animal Health, University of South Africa, Roodepoort, Johannesburg 1710, South Africa; ^4^Department of Veterinary Physiology and Biochemistry, Faculty of Veterinary Medicine, University of Ibadan, Ibadan, Oyo, Nigeria; ^5^Department of Veterinary Physiology and Pharmacology, College of Veterinary Medicine, Michael Okpara University of Agriculture Umudike, Umuahia, Abia, Nigeria

**Keywords:** apoptosis, cadmium, inflammation, PREOG, q-RT-PCR, testicular activity

## Abstract

Cadmium, as a toxic heavy metal abounds in our habitat, and its impact on the testes contribute to the global decrease in the male fertility rate. Several natural compounds have been used to manage Cd-induced infertility successfully. Polyphenol-rich extract of *Ocimum gratissimum* is one plant whose potency has been reported in various studies. Nevertheless, no report has explored PREOG impairment of reprotoxicity induced by cadmium in male animals. This study, therefore, is designed to evaluate the effect of PREOG against cadmium reprotoxicity in adult male Wistar rats. 66 male rats were randomly assigned to 6 groups A to F (*n* = 11) per group, orally received the following treatment daily for 8 weeks: A (distilled water alone as control), B (3 mg/kg CdCL_2_), C (100 mg/kg PREOG), D (200 mg/kg PREOG), E (100 mg/kg PREOG + 3 mg/kg CdCL_2_), and F (200 mg/kg PREOG + 3 mg/kg CdCL_2_) at the end, six rats from each group were sacrificed, blood, semen, and testes were harvested for analysis, while the remaining males from each group were introduced to untreated female in a ratio of 2:1 for fertility study and result indicated; PREOG treatment enhances testicular weight, GSI and EPI, sperm quality and quantity, serum testosterone levels, and upregulate steroidogenic gene (3β-HSD, 17β-HSD, and StAR) expression. PREOG impaired testicular oxidative stress (enhance GST, SOD, and GPx value) and inflammation by decreasing testicular expression of COX-2 and TNF-α, and plasma IL-6 and TNF-α concentration. Also, PREOG impaired testicular apoptosis by decreasing caspase-3 protein distribution, protected histopathologic alteration of the testes, and finally enhanced reproductive outcome. The impairment of cadmium-induced reprotoxicity in male rats by PREOG treatment affirms its therapeutic and reproductive benefits.

## 1. Background

Reproduction is an indispensable activity that guarantees the progression of species and their heritable irregularity. A successful reproductive outcome entails the differentiation and production of gametes of high quality that can fertilize and be fertilized. The male gamete also known as the spermatozoa is very unique cells that endure close biological and structural changes through spermatogenesis and epididymal movement [1, 2]. These processes are delicate and are prone to errors that can impair sperm quality. The recent global decline in fertility rate is associated with the depreciating quality of both the male and female gametes [3].

With the rising trend of infertility in men and animals, several advances have been made in the diagnostic and therapeutic management of infertility cases in humans, yet the global human population between 1990 and 2010 was almost static, recording a 0.1% decline in primary infertility rate and 0.4% increase in secondary infertility rate [4]. In animals, even though the scale of studies is limited, the available information shows subfertility is rising in livestock, due to increased culling rates and delayed calving intervals consequently impeding animal welfare and the farm economy. Also, the authors of [5] documented a decline in the pregnancy rate in dairy cattle after the first service over the past 30–50 years. Naturally, males and females contribute to infertility cases. But before now, little emphasis is paid to the contribution of males to conception, embryo health, and early childhood growth. Recent findings also show that 40%–50% of all infertility cases are a result of male factors [6, 7].

Infertility in males is the inability to achieve pregnancy (childlessness) with a fertile female [8], and it could result from numerous factors such as impaired production, transport, or storage of spermatozoa; altered libido and incomplete or complete inability to mate [9], changes in hormonal levels, sexual dysfunction, untreated sexually transmitted diseases, varicocele, and sperm DNA damage can result to male infertility [10]. Furthermore, anatomical abnormalities [11], lifestyle [12, 13], and environmental stressors also impair male fertility [14, 15]. Since the release of these pollutants is continuous, exposure of man and animal is inevitable/unavoidable; thus, intoxication occurs as a result of prolonged exposure [16, 17].

Numerous environmental pollutants have been documented to impair the testes deleteriously, some mimic vital physiological ions, thus interfering with normal biological processes such as the endocrine system. These compounds are referred to as endocrine-disrupting compounds (EDCs) such as heavy metals (e.g., cadmium) [18–20]. Water and food are the primary routes, humans and animals are intoxicated by cadmium in a population of nonsmokers [21–23], and cadmium is 4-5 times higher in the blood of those that smokers in contrast to those that do not [24]. It is obvious from studies that the testes remain one vital organ affected by Cd intoxication, and this accounts for the recent decline in the male fertility rate [25]. Cadmium has been reported to impair testicular activity at various levels, such as steroidogenesis [26, 27], the blood-testis barrier integrity [28, 29], the seminiferous epithelium cytoarchitecture integrity [30–32], and sperm parameters, especially motility [29, 33].

Even though the causative factors or etiology that culminates in the decline of the male fertility rate is multidimensional, a common intrinsic mechanism of impairing the complex balance of ROS production and scavenging is shared by most of these factors. This imbalance reduces antioxidant capacity and increases ROS generation leading to oxidative stress (OS) [34]. The defiance of this redox imbalance is a major threat the spermatozoa must contend with, and in most cases, the imbalance between free radicals (reacting nitrogen and oxygen species (RNS/ROS)) and the antioxidant system favors the oxidants [35]. While the numerous physiological functions of the spermatozoa are regulated by redox metabolism, such as sperm-oocyte interactions, capacitation, and acrosome reaction (AR) [36, 37], unlimited generation or elevated number of detrimental radicals to all cell components and at molecular level, DNA and lipids are specifically vulnerable [36]. The damage of the spermatozoa membrane by lipid peroxidation can deter its fluidity resulting in impaired acrosome and capacitation reaction (AR), and the mitochondria membrane, resulting in dysfunction and ATP synthesis decline [37]. Furthermore, the paternal contribution to embryo genomics is seriously compromised as a result of damage to the spermatozoa DNA [38, 39]. In normal spermatozoa, OS may result from the presence of leukocytes that generate ROS in the semen exposure to exogenous substances, spermatozoa mitochondria metabolism, and in extended semen for assisted reproductive technique (ART) [36]. In regards to this several studies using different antioxidant compounds to manage the reprotoxic effects due to cadmium intoxication have been documented by [14, 40].

Polyphenolic-rich extract of *Ocimum gratissimum* (PREOG) is a fraction of *Ocimum gratissimum* rich in phytocompounds such as flavonoids, phenol, and tannin. Invitro assay of PREOG shows it a very powerful antioxidant with an IC_50_ below 50 [41] and the therapeutic properties of Ocimum gratissimum can be associated with its rich phenolic content which is ascribed to its strong antioxidative property it possesses [42, 43]. The polyphenolic extract of *Ocimum gratissimum* has been used in several studies to manage different pathologic conditions [44–47]. Of the numerous works done with various fractions of *Ocimum gratissimum*, very scarce studies documented the ameliorating effects of PREOG against cadmium-induced reprotoxicity in any specie in an in-vivo or in-vitro study. Therefore, the current study focuses on the antagonistic effect of PREOG against cadmium-induced reprotoxicity, emphasizing gonadosomatic index (GSI), epididymal index (EPI), histopathological alterations, oxidative function, inflammation, apoptosis, semen quality, steroidogenesis, and fertility study in adult male rats.

## 2. Materials and Methods

### 2.1. Plant Collection and Identification

Freshly harvested leaves of *Ocimum gratissimum* were obtained from different farmers around Ibadan, Oyo State, Nigeria. The harvested plant was authenticated after identification at the Botany Department, University of Ibadan, Ibadan. A certificate sample was stored at the herbarium with reference number UIH-22617.

### 2.2. Polyphenolic Extraction

The leaves were dried in a shaded area and made into their powdered form. The fat in the powder was removed by immersing it for 24 h in n-hexane. The nonfat powder is soaked in methanol for another 72 h to get the methanol crude extract. The crude methanol extract was fortified (concentrated) using a rotatory evaporator at 40°C. The sticky residue was then segmented into chloroform soluble fractions using chloroform. The chloroform-rich fraction was then evaporated and dried in an oven under reduced pressure to obtain a polyphenolic fraction of *Ocimum gratissimum* (PREOG) [47]. The concentrations 100 and 200 mg/kg were chosen following the result we got from our toxicity study.

### 2.3. Reagents

Cadmium chloride was purchased from Loba chemicals in Oyo State, Ibadan as cadmium chloride monohydrate (98%) extra pure, CAS number 35658-65-2 was reconstituted in distilled water to obtain a concentration of 3 mg/kg with slight modification [48]. 100% Wesson corn oil was used to reconstitute our extract before administration.

### 2.4. Experimental Animals

Sixty-six (8–10) weeks adult male Wistar rats weighing (200–210) g, were acquired from a commercial breeder in collaboration with the Faculty of Veterinary Medicine, University of Ibadan, Nigeria. In a well-aerated animal house, plastic cages, and exposed to 12 h light-dark cycle the rats were monitored. Feeding was done with commercial rat chow and water was always accessible. Animal handling was done humanely following the guidelines/criteria for managing laboratory animal use and care [49] and recommendations. Also, the rats were managed using recommendations of the University of Ibadan Animal Care and Use Research Ethics Committee. All the techniques were reviewed and assented to by the committee with the allotted number UI-ACUREC/016-0122/24.

### 2.5. Experimental Design (Phase One)

The sixty-six adult male Wistar rats were randomly assigned to six groups A–F of eleven rats each. Group A was only allowed distilled water ad libitum as the positive control, group B received 3 mg/kg of cadmium alone as negative control it is 1/15 of the oral LD50 values in rats [48], C received 100 mg/kg PREOG alone, D received 200 mg/kg PREOG alone, E received 3 mg/kg cadmium and 100 mg/kg PREOG, and F received 3 mg/kg cadmium and 200 mg/kg PREOG. The treatment was done orally and lasted for 61 days to cover the spermatogenic cycle of adult rats [50, 51]. At day 62, six rats from each group were sacrificed. The blood collected from the retro-orbital sinus, semen from the caudal epididymis, and the harvested testes were used for analysis.

#### 2.5.1. GSI and Epididymis Index

Using a sensitive electronic weighing machine, weights of the right/left testes/epididymis were immediately measured. GSI and EPI were computed as the average testicular weight (g) or epididymal weight (g) divided by the weight difference (at day one and sixty-five) (g) and multiplied by 100. GSI and EPI value is expressed in percentage (%) [52].

#### 2.5.2. Motility

Sperm motility was determined using procedures described by Zemjanis [53] with slight modification by [54]. Semen was isolated from the caudal epididymis and placed in a warm slide. Using a warm sodium citrate buffer (2.9%) the semen was mixed, and a cover slip was then placed on the slide before microscopic evaluation. From isolation to examination is approximately 2–4 min and our result is expressed in percentage.

#### 2.5.3. Livability

A Smears was prepared from the caudal epididymal semen in a nongreasy slide and stained with Wells and Awa 1% Eosin and 5% Nigrosin in 2.9% sodium citrate dehydrates solution for the live/dead ration determination as demonstrated by [54] with slight modification.

#### 2.5.4. Sperm Count

Epididymal sperm count was evaluated by chopping up the cauda epididymis in distilled water and sieve with a nylon mesh. The spermatozoa were counted using Pant and Srivastava improved Neubauer chamber technique as demonstrated by [54] with slight modification.

#### 2.5.5. Morphological Characteristics

A smear is made on a clean slide free of grease with Semen collected from the caudal epididymis and stained with Awa and Wells stain as demonstrated by [54]. The smear was dried in air and with the aid of a microscope the abnormal cells from several fields of at least 400 sperm cells were counted and their total percentage was determined.

#### 2.5.6. Serum Hormonal Assay

Serum harvested was transferred into plain bottles for analyzing luteinizing hormone (LH), testosterone (T), and follicle-stimulating hormone (FSH) levels using standard enzyme-linked immunosorbent assay (Monobind ELISA) kits (425-300 FSH AccuBind, 3725-300 testosterone AccuBind, 675-300 LH AccuBind) following the instructions of the manufacturers.

#### 2.5.7. Serum Interleukin 6 (IL-6), and Tumor Necrosis Factor (TNF-α)

Serum levels of IL-6 and TNF-α were determined using commercially purchased Enzyme-linked immunosorbent Elabscience (ELISA) assay kits (Cat No: E-EL-R2856 for TNF-α and Cat No: E-EL-R0015 for IL-6), following the instructions of manufacturers. The concentrations of the cytokines were determined and expressed as picograms per mL.

#### 2.5.8. Tissue Processing

##### 2.5.8.1. Tissue Biochemical Assays

The testes using 50 metros'–HCl buffer (pH 7.4) containing 1.15% potassium chloride were homogenized and the homogenate was centrifuged for 15 min at 12,000 rpm and 4°C. The resulting supernatant was used to estimate biochemical parameters. Total protein concentration was as documented [55]. Hydrogen peroxide yielded was as reported [56]. The malondialdehydes (MDA) value was demonstrated as described [57]. Superoxide dismutase (SOD) is documented [58] with little remodeling by [59]. Glutathione S-transferase (GST) was determined as documented [60]. Glutathione peroxidase (GPx) activity was carried out as documented [61], where hydrogen peroxide was used as substrate. Reduced glutathione (GSH) was determined as demonstartede by [62] at 412 nm, with little modifications [59].

##### 2.5.8.2. Quantitative Real-Time PCR

Total RNA extraction from the testes of the experimental group was done by modified CTAB extraction protocol to dissipate extracted DNA, and 20 ng of total RNA was treated with NEB DNase 1 (M0303). For the gene quantification, 20 μL reactions following the manufacturer's guidelines using Luna Universal qPCR Master Mix Protocol (M3003) to identify the presence of StAR, 3β-HSD, and 17β-HSD genes from the RNA extracted. Actin gene expression was the control. The Luna Universal qPCR Master mix used was 10 μL, 0.06 reverse transcriptase (Promega) was made to 18 μL with nuclease free water, of which 2 μL of the treated RNA, 0.5 μL of the forward primer (10 μM), and 0.5 μL reverse primer (10 μM) were added. This was then run with the outline starting denaturation for 60 s at 95°C, then 40–45 of denaturation for 15 s at 95°C, plate reading, and extension for 30 s at 60°C, and termination for 10 min at 72°C. Amplification was carried out with a cfx96tm real-time system from Bio-Rad using their manual. The threshold cycle number (Ct value) was determined with the cfx96tm real-time system from Bio-Rad. QPCR was standardized to the Ct value of ACTB, and the same sample fold change for each gene was computed using the delta-delta CT procedure. A nontemplate control was included in each experiment. Primer sequences used for qRT-PCR of genes are indicated in the table below and are synthesized in South Africa at Inqaba (Table 1).

##### 2.5.8.3. Immunohistochemistry

The procedure for the expression of Caspase 3 (Casp-3), cyclooxygenase-2 (COX-2), and TNF-α in the testes was determined following standard protocols (with little modification) as demonstrated [63] using with DAB solution a 2-step plus Poly-HRP Anti-Mouse/Rabbit IgG Detection System (catalog number: E-IR-R217 from Elabscience Biotechnology, China). Fixing of testes was done with 10% paraformaldehyde before embedment and sectioning using a thickness of 5 μm. 100% xylene was then used to deparaffinized the slides, and this was followed immediately by hydration with 100%, 90%, 80%, and 70% ethanol for 2 min in each concentration, and then, slides were placed in a tank of PBS for 5 min. Antigen was retrieved via citrate buffer (14.75 g trisodium citrate dehydrate and 2.1 g citric acid monohydrate) pH 6.0, in a microwave oven 2 to 3 times. The stained slides were then covered with drops of endogenous peroxide (H_2_O_2_ block) from the manufacturer's kit (E-IR-217C) before incubation at room temperature for 10 mins. Afterward, the slides were rinsed and put back in the PBS tank for 5 min. Nonspecific binding was impeded in the slide by adding goat serum (E-1R-R217A) and incubated in a humidifying chamber for 30 min at room temperature. Afterward, the tissues were probed with the following: COX-2 polyclonal antibody (E-AB-17010: 1:100 dilution), caspase-3 polyclonal antibody (E-AB-22213: 1:150 Dilution), and TNF-α polyclonal antibody, after incubation in a humidifying chamber for 2 h at room temperature. Then, we rinse the slide with PBS and a secondary antibody labeled (E-1R-R217B) and incubate again in the humidifying chamber for 20 min at room temperature. Finally, slides were rinsed again before a few drops of diaminobenzidine (DAB) solution (50 μL of DAB concentrate (E-1R-R217D), and 1 mL DAB substrate (E-1R-R217E) prepared in the dark were added at room temperature for 10 s. Using double-distilled or deionized water, the reaction was terminated, and slides were inserted for 3 s in hematoxylin before rinsing with PBS. Slides were then placed for 1 min each in the following ethanol concentrations (70%, 80%, 90%, and 100%) and 100%xylene. Slides were removed and allowed to dry, and a DPX mountant was applied. Sections were viewed with a light microscope (Leica LAS-EZ) using the Leica software application suite version 3.4 equipped with a digital camera.

##### 2.5.8.4. Histological Preparations

The testes harvested from each rat were fixed by immersing it in a Bouin's solution for 48 h. Afterward, the fixed testes were dehydrated using ethanol with different concentrations, and xylene was used to clear before embedment in paraffin wax. A section of 5 μm thick was excised and mounted on glass slides. Staining was done using eosin and hematoxylin before examination with the aid of compound light microscopy with an attached digital camera.

### 2.6. Fertility Study (Phase Two)

Two weeks before the conclusion of phase one, 60 adult female albino Wistar rats weighing 150 ± 10 g were purchased and allowed to acclimatize. They were exposed to the treated male of phase one in a ratio of two females to a male (2:1) for ten days before the males were removed and the females kept in their separate cages until term. The fertility percentage was computed from the breaded females and the number that conceive. The females that were pregnant were allowed to have their pups. Females that conceived in each group and the number of pups born were computed and indicated as mean values of each group, and the viability of pups from PND0 to PND7 was determined as the survival index for each litter, and the fertility indices were decided according to the procedure of [64, 65] with slight adjustment as follows.

#### 2.6.1. Male Fertility Index

(1) $$\begin{array}{c} \text{Fertility index}=\left(\frac{\text{fertile male}}{\text{total number of males used for study}}\right)\times 100. \end{array}$$

#### 2.6.2. Female Fertility Index

(2) $$\begin{array}{c} \text{Fertility index}=\left(\frac{\text{pregnant female}}{\text{total number of females used for the study}}\right)\times 100. \end{array}$$

#### 2.6.3. The Parturition Index

(3) $$\begin{array}{c} \text{Parturition index}=\left(\frac{\text{females that delivered their pups}}{\text{number of pregnant rats}}\right)\times 100. \end{array}$$

#### 2.6.4. The Gestation Index

(4) $$\begin{array}{c} \text{Gestation index}=\left(\frac{\text{pups burn alive}}{\text{total number of pups burn}}\right)\times 100. \end{array}$$

The total number of offspring delivered was indicated as the mean percentage of delivered offspring at birth.

### 2.7. Statistical Analysis

Data obtained from this investigation were presented with mean ± SD. Applying one-way ANOVA, the mean difference across the various groups was determined. Post hoc analysis to decide the difference between two groups was determined using the Student' *t*-test and Tukey's test. All analyses were performed using GraphPad Prism version five. *p* < 0.05 was treated as statistically significant.

## 3. Result

### 3.1. The GSI and EPI, and Some Testicular Biometry Posttreatment With PREOG/Cadmium

In Table 2, the body weight gain in the treated male rats differs significantly (*p* < 0.05) across all groups. When contrasted to control, body weight gain differs notably (*p* < 0.05) in the cadmium group. Co-treatment of different doses of PREOG and cadmium had a notable effect on weight gain (*p* < 0.05) in contrast to the cadmium group and the control group (Table 2). The average testicular weight and epididymal weight were comparable across all groups. In contrast to the control, average testicular weight decreased notably (*p* < 0.05) in the cadmium group, while the epididymal weight was insignificant (*p* > 0.05) in the cadmium group. Co-treatment with different doses of PREOG mitigated the cadmium effect and notably increased the average testicular weight (*p* < 0.05) when contrasted to the cadmium group, but the average testicular weight was insignificant (*p* > 0.05) from the control. However, in group F (1.09 ± 0.43), average testicular weight is comparable to the cadmium group B (1.01 ± 0.19), while in group E (1.20 ± 0.08), average testicular weight differs notably (*p* < 0.05) from the control A (1.34 ± 0.11). Co-treatment with different doses of PREOG and cadmium had an insignificant effect on epididymal weight (*p* > 0.05) in contrast to cadmium and control groups (Table 2).

Using the body weight gain, average testicular, and epididymal weight, the GSI and EPI were determined. From our result, EPI and GSI values differ significantly (*p* < 0.05) in all groups. In contrast to the control, EPI and GSI values decrease significantly (*p* < 0.05) in the cadmium group. Co-treatment with different doses of PREOG abrogated the cadmium effect and significantly increased EPI and GSI values (*p* < 0.05) in contrast to the cadmium group, but the increase was insignificant (*p* > 0.05)when contrasted to the control. However, in group E (1.34 ± 0.1), EPI value differs significantly (p ˂ 0.05) from the control A (1.08 ± 0.1). Furthermore, the treatment with different doses of PREOG alone had a notable effect on EPI and GSI values (*p* < 0.05) when contrasted to the control and cadmium groups. However, the EMI value in group D (1.81 ± 0.2) was insignificant (*p* > 0.05) to cadmium group B (0.83 ± 0.1) (Table 2).

The right/left testicular diameter/length was insignificant (*p* > 0.05) across all groups. In contrast to the control, the right/left testicular diameter/length was insignificant (*p* > 0.05) in the cadmium group. Co-treatment with different doses of PREOG and cadmium also had an insignificant effect on the right/left testicular diameter/length (*p* > 0.05) in contrast to the cadmium group and control group (see Table 2).

### 3.2. The Sperm Quality and Morphology Posttreatment With PREOG/Cadmium

Table 3shows the impact of PREOG on the semen quality in adult male Wistar rats exposed to cadmium. The sperm motility, livability, and count differ notably (*p* < 0.05) in all groups. In contrast to the control group, sperm motility, livability, and count significantly decreased (*p* < 0.05) in the cadmium group. Co-treatment with different doses of PREOG abrogated the cadmium effect and increased the sperm motility, livability, and count notably (*p* < 0.05) in contrast to the cadmium group, and the increase was also notably different (*p* < 0.05) from the control. Furthermore, the treatment with different doses of PREOG alone significantly increases sperm motility, livability, and count (*p* < 0.05) in contrast to the control and cadmium groups (Table 3).

The sperm's total morphological abnormality was evaluated using the head, middle, and tail defects. In our result, the head, middle, and tail defects differ significantly (*p* < 0.05) in all groups. In contrast to the control group, the head, middle, and tail defects increase significantly (*p* < 0.05) in the cadmium group. Co-treatment with different doses of PREOG abrogated the cadmium effect and decreased the head, middle, and tail defects (*p* < 0.05) significantly in contrast to the cadmium group, and the decrease was also notably different (*p* < 0.05) from the control group. However, the head defect in all treated groups was comparable to the control group, and the middle defect in group E (6.16 ± 1.86) was also comparable to control A (5.89 ± 0.85). Additionally, the treatment with different doses of PREOG alone decreased the head, middle, and tail defects significantly (*p* < 0.05) from the control and cadmium groups (Table 3). Total morphological abnormality was markedly different (*p* < 0.05) across all groups. In contrast to the control group, total morphological abnormality increased significantly (*p* < 0.05) in the cadmium group. Co-treatment with different doses of PREOG abrogated the cadmium effects and decreased the total morphological abnormality markedly (*p* < 0.05) from the cadmium group, and the decrease was also notable (*p* < 0.05) from the control group. However, in group E (12.27 ± 3.62), total morphological abnormality was insignificant (*p* > 0.05) to control A (10.08 ± 0.82) (Table 3).

### 3.3. Serum Testosterone, FSH, and LH Values Posttreatment With PREOG/Cadmium

**Figure 1:**
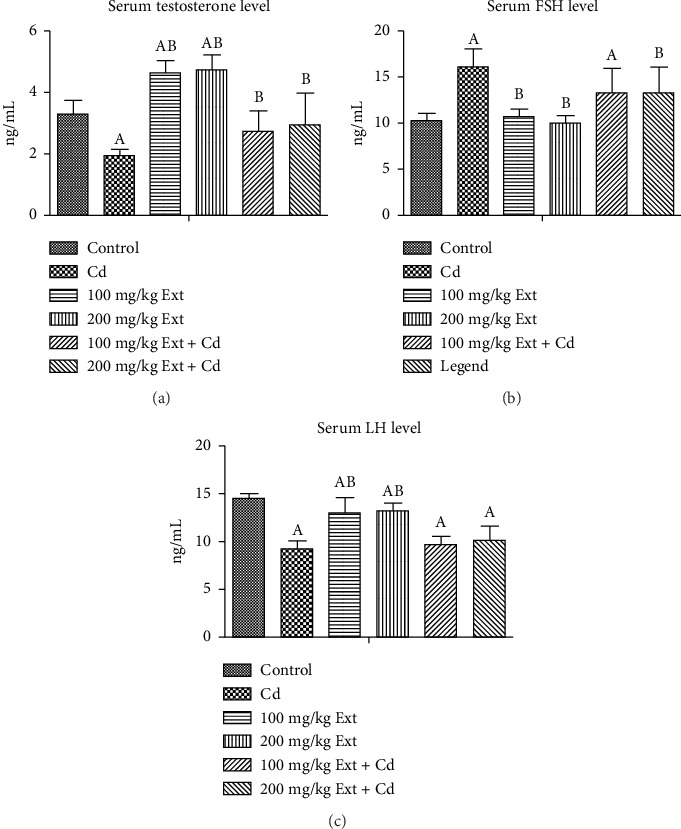
a, b & c. Serum testosterone and FSH and LH levels in adult Wistar rats treated with Cd and PREOG. Values are expressed in mean ± SD. Superscripts (A & B) indicate a notable difference from the control and cadmium groups. Abbreviations: Cd, CdCL2; Ext, PREOG; FSH, follicle-stimulating hormone; LH, luteinizing hormone.

Figure 1(a) Shows serum testosterone level of adult male Wistar rats exposed to PREOG and cadmium. From our result, serum testosterone value was significantly different (*p* < 0.05) in all groups. The testosterone value was significantly decreased (*p* < 0.05) in the cadmium group in contrast to the control group. The co-treatment with different doses of PREOG abrogated the cadmium effect and increased serum testosterone levels significantly (*p* < 0.05) from the cadmium group, but this increase was insignificant (*p* > 0.05) from the control group. Treatment with different doses of PREOG alone was insignificant (*p* > 0.05) to the control group but differs notably (*p* < 0.05) from the cadmium group.

Figure 1(b) shows the serum FSH level of adult male rats treated with PREOG and cadmium. This figure indicated a significant difference (*p* < 0.05) in the serum (FSH) value across all groups. In contrast to the control, the FSH level increases significantly (*p* < 0.05) in the cadmium group. The different doses of PREOG decreased the FSH value significantly (*p* < 0.05) from the cadmium group, but the decrease was insignificant (*p* > 0.05) to control group. However, in the 100 mg/kg PREOG plus cadmium-treated group, the FSH value was significantly different (*p* < 0.05) from the control group. Additionally, the treatment with different doses of PREOG alone on FSH value differs significantly (*p* < 0.05) from the cadmium group but was insignificant (*p* > 0.05) to control group.

Figure 1(c) Shows (*p* < 0.05) across all groups. In contrast to the control group, serum LH levels decrease significantly (*p* < 0.05) in the cadmium group. Co-treatment with different doses of PREOG and cadmium had an insignificant effect on serum LH value (*p* > 0.05) in contrast to the cadmium group, but in contrast to control group, LH value differs significantly (*p* < 0.05). Furthermore, the LH value after treatment with different doses of PREOG alone was significantly different (*p* < 0.05) from the control and cadmium groups (Figure 1(c)).

### 3.4. Effects of Oral PREOG/Cadmium on 3β-HSD, 17β-HSD, and StAR Steroidogenic Gene

**Figure 2:**
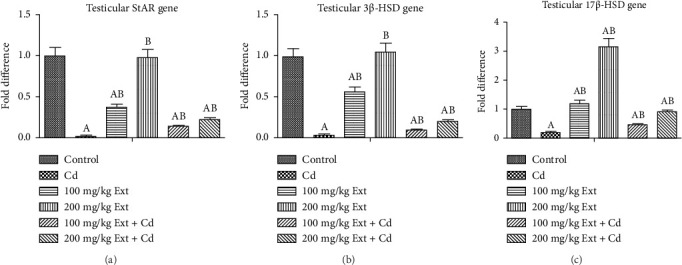
a, b & c. Testicular StAR, 3β-HSD, and 17β-HSD genes expression in adult Wistar rats treated with Cd and PREOG. Values are expressed in mean ± SD. Superscripts (A & B) indicate a notable difference from the control and cadmium groups. Abbreviations: Cd, CdCL2; Ext, PREOG; StAR, steroidogenic acute regulatory proteins, and 3 & 17β-HSD, hydroxysteroid dehydrogenase.

Figures 2(a), 2(b), and 2(c) show the testicular steroidogenic (StAR, 3β-HSD, and 17β-HSD) gene following the treatment of adult male Wistar rats with cadmium and PREOG using qRT-PCR. From our result, in the cadmium group, there was a notable downregulation of StAR, 3β-HSD, and 17β-HSD gene expression (*p* < 0.05) when contrasted to control group. The co-treatment with different doses of PREOG abrogated the cadmium effects and upregulated the steroidogenic genes (StAR, 3β-HSD, and 17β-HSD) expression notably (*p* < 0.05) in contrast to the cadmium and control groups. Additionally, the treatment with different doses of PREOG alone upregulates StAR, 3β-HSD, and 17β-HSD gene expression significantly (*p* < 0.05) in contrast to the control and cadmium groups. However, in the 100 mg/kg PREOG treated group, the 3β-HSD gene was downregulated significantly (*p* < 0.05) in contrast to control (Figure 2(b)), and StAR gene was also significantly downregulated (*p* < 0.05) in 100 mg/kg PREOG treated group in contrast to control (Figure 2(a)).

### 3.5. The Oxidative Status Posttreatment With PREOG/Cadmium

Table 4 shows the oxidative status in adult male Wistar rats treated with PREOG and cadmium. The hydrogen peroxide (H_2_O_2_) value differs significantly (*p* < 0.05) in all groups. In contrast to the control group, the H_2_O_2_ value increase was statistically significant (*p* < 0.05) in the cadmium group. Co-treatment with different doses of PREOG mitigated the cadmium effects and decreased the H_2_O_2_ value notably (*p* < 0.05) from the cadmium group, and the decrease also differs notably (*p* < 0.05) from the control group. Furthermore, the H_2_O_2_ value of different doses of PREOG alone treatment was insignificant (*p* > 0.05) in contrast to control group but significant (*p* < 0.05) in contrast to the cadmium group (Table 4).

The reduced glutathione (GSH) value differs notably (*p* < 0.05) across all groups. In contrast to the control group, the GSH value decreases significantly (*p* < 0.05) in the cadmium group. Co-treatment with different doses of PREOG mitigated the cadmium effects, increasing the GSH value comparably to the cadmium group, but the GSH value differs significantly (*p* < 0.05) from the control group. Additionally, the GSH value in the different doses of PREOG alone treatment was statistically insignificant (*p* > 0.05) to control group, but there was a significant difference (*p* < 0.05) to the cadmium group (Table 4).

Lipid peroxidation was determined from the malondialdehyde (MDA) values which from our result was significantly different (*p* < 0.05) in all groups. In contrast to the control, the MDA value increased significantly (*p* < 0.05) in the cadmium group. Co-treatment with different doses of PREOG abrogated the cadmium effects and decreased the MDA values (*p* < 0.05) significantly from the cadmium group, but in contrast to control group, MDA value was insignificant (*p* > 0.05). However, in group F (7.97 ± 4.65), MDA value was insignificant (*p* > 0.05) in the cadmium group B (13.09 ± 4.17). Additionally, the MDA values of the different doses of PREOG alone treatment were statistically insignificant (*p* > 0.05) to control group, but in contrast to the cadmium group, MDA value differs significantly (*p* < 0.05) (Table 4).

The SOD and GPx and GST value differs notably (*p* < 0.05) in all groups. In contrast to the control group, there was a notable decrease in the GPx, SOD, and GST values (*p* < 0.05) in the cadmium group. Co-treatment with different doses of PREOG abrogated the cadmium effects, increasing GPx and SOD values significantly (*p* < 0.05) from the cadmium group, and the increase also differs significantly (*p* < 0.05) to control group. However, in group F (65.98 ± 7.08), the GPx value was insignificant (*p* > 0.05) in contrast to cadmium group B (49.09 ± 8.92). The co-treatment of PREOG with cadmium did not exert any notable effect on GST value (*p* > 0.05) when contrasted to cadmium and control groups. However, in group E (19.01 ± 2.07), GST value differs notably (*p* < 0.05) from control A (26.43 ± 4.67). Furthermore, the GPx, SOD, and GST values of the different doses of PREOG alone treatment were statistically insignificant (*p* > 0.05) to the control group, but when contrasted to the cadmium group, there was a notable difference (*p* < 0.05) (see Table 4).

### 3.6. Inflammatory Markers Posttreatment With PREOG/Cadmium

**Figure 3:**
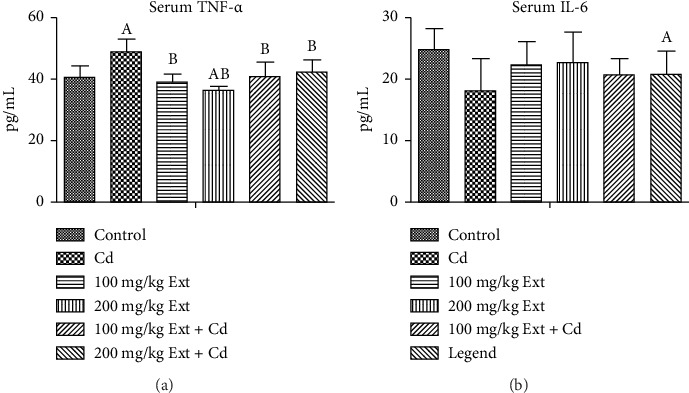
a and b. Serum TNF-α and IL-6 value in adult Wistar rats treated with Cd and PREOG. Values are expressed in mean ± SD. Superscripts (A & B) indicate a notable difference from the control and cadmium groups. Abbreviations: Cd, CdCL2; Ext, PREOG; TNF-α, tissue necrotic factor alpha; IL, interleukin.

Figures 3(a) and 3(b) show the serum inflammatory markers in adult male rats exposed to PREOG and cadmium. Serum tissue necrotic factor alpha (TNF-α) value differs notably (*p* < 0.05) across all groups. In contrast to the control group, the serum TNF-α value differs notably (*p* < 0.05) in the cadmium group. Co-treatment with different doses of PREOG mitigated the cadmium effects and decreased serum TNF-α values significantly (*p* < 0.05) from the cadmium group, but the decrease was insignificantly (*p* > 0.05) to the control group. Additionally, the serum TNF-α value for the different doses of PREOG alone treatment was statistically insignificant (*p* > 0.05) when contrasted to the control, but in contrast to the cadmium group, there was a notable difference (*p* < 0.05) (Figure 3(a)). There was an insignificant difference (*p* > 0.05) of serum IL-6 level across all groups. Cadmium treatment did not have any notable effect on serum IL-6 level (*p* > 0.05) in contrast to the control. Co-treatment with different doses of PREOG also had an insignificant effect on serum IL-6 Level (*p* > 0.05) from the cadmium and control groups. Treatment with different doses of PREOG alone did not notably affect serum IL-6 level (*p* > 0.05) when contrasted to control, but there was a notably decrease (*p* < 0.05) from the cadmium group (Figure 3(b)).

### 3.7. Effects of Oral PREOG Leaves/Cadmium on the Fertility Index of Adult Male Rats

Table 5 indicates the effect of oral PREOG on fertility in adult male Wistar rats treated with cadmium. From our result, the fertility index of adult males treated with cadmium, the parturition, and the gestation index decreased by 50% in the cadmium group when contrasted to the control. Cotreatment with different doses of PREOG abrogated the cadmium effects and enhanced by 30% the male/female fertility index and the parturition/gestation index in contrast to the cadmium group, but the increase was 20% less when contrasted to the control group. Additionally, the treatment with different doses of PREOG alone had an insignificant effect on the male/female fertility index and the gestation/parturition index when contrasted to the control group, but when compared to the cadmium group, there was a 50% increase in the male/female fertility index and gestation/parturition index (Table 5).

The percentage of pups delivered (%) differs notably (*p* < 0.05) in all groups. In contrast to the control, % pups delivered decreased significantly (*p* < 0.05) in the cadmium group. Cotreatment with different doses of PREOG abrogated the cadmium effect and increased the % of pups delivered significantly (*p* < 0.05) in contrast to the cadmium and control groups. However, in group F (5.6 ± 1.5)%, the pups delivered were insignificant (*p* > 0.05) in contrast to cadmium group B (5.6 ± 0.6). Furthermore, the treatment with different doses of PREOG alone had an insignificant effect on % pups delivered (*p* > 0.05) in contrast to the control, but in contrast to the cadmium group, % pups delivered were notable (*p* < 0.05) (Table 5).

The birth weight was insignificant (*p* > 0.05) across all groups. In contrast to control, birth weight was also not notable (*p* > 0.05) in the cadmium group. Cotreatment with different doses of PREOG and cadmium had an insignificant effect on birth weight gain (*p* > 0.05) in contrast to the cadmium and control groups. Additionally, the treatment with different doses of PREOG alone also had an insignificant effect on weight gain (*p* > 0.05) compared to the control and cadmium groups (Table 5).

The survival index of the pups for the first 7 days after parturition was insignificant (*p* > 0.05) across all groups. In contrast to control, the survival index was insignificant (*p* > 0.05) in the cadmium group. Cotreatment with different doses of PREOG and cadmium did not have any marked effect on the survival index (*p* > 0.05) when contrasted to the cadmium and control groups. Furthermore, the treatment with different doses of PREOG alone also had an insignificant effect on the survival index (*p* > 0.05) when contrasted to the control and cadmium groups (Table 5).

### 3.8. Immunohistochemistry of the Testes Posttreatment With PREOG/Cadmium

**Figure 4:**
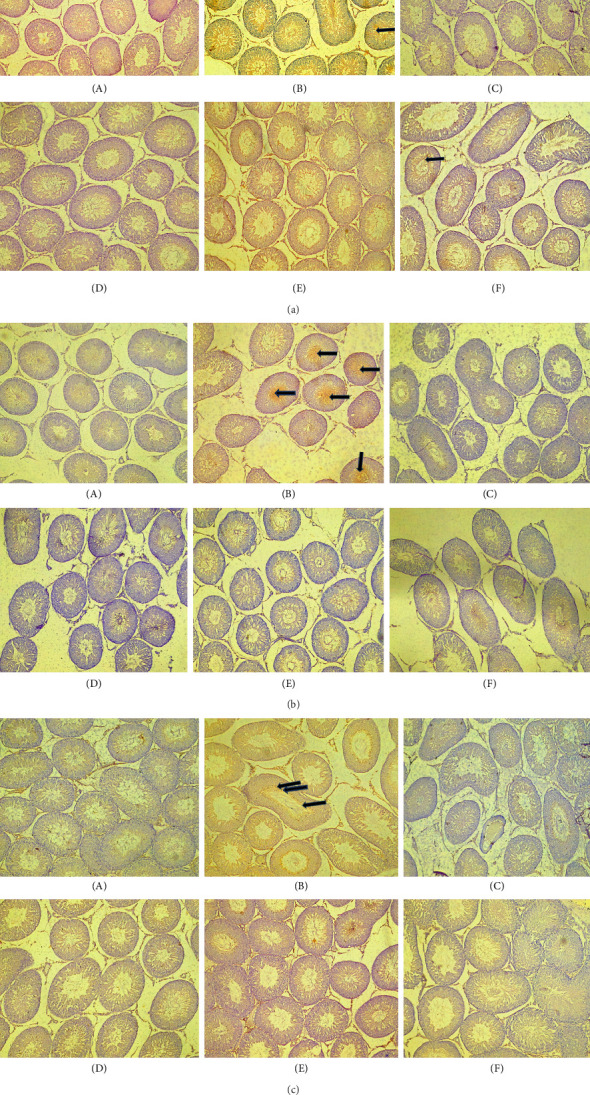
(a) Immunohistochemistry for testicular caspase-3. A (control) in adult Wistar rats treated with cadmium and PREOG. B (3 mg/kg CdCL), C (100 mg/kg PREOG), D (200 mg/kg PREOG), E (100 mg/kg PREOG + 3 mg/kg CdCL), and F (200 mg/kg PREOG + 3 mg/kg CdCL). Slides were stained with high-definition hematoxylin (×40). (b) Immunohistochemistry for testicular COX-2. A (control) in adult Wistar rats treated with cadmium and PREOG. B (3 mg/kg CdCL), C (100 mg/kg PREOG), D (200 mg/kg PREOG), E (100 mg/kg PREOG + 3 mg/kg CdCL), and F (200 mg/kg PREOG + 3 mg/kg CdCL). Slides were stained with high-definition hematoxylin (×40). (c) Immunohistochemistry for tissue necrotic factor (TNF-α) in the testes of adult Wistar rats treated with Cd and PREOG. A (control), B (3 mg/kg CdCL), C (100 mg/kg PREOG), D (200 mg/kg PREOG), E (100 mg/kg PREOG + 3 mg/kg CdCL), and F (200 mg/kg PREOG + 3 mg/kg CdCL). Slides were stained with high-definition hematoxylin (×40).

Figures 4(a), 4(b), and 4(c) show a photomicrograph of changes in caspase-3, COX-2, and TNF-α expression in the testicular tissue of adult Wistar rats following treatment with various doses of PREOG and cadmium (magnification, × 400). In Figure 4(a), immunohistochemical analysis clearly showed an intensified signal for caspase-3 in the seminiferous tubular cells of the cadmium-treated group. The different doses of PREOG repudiate the cadmium effects and decrease significantly the number of caspase-3 positively stained seminiferous tubular cells when contrasted to the cadmium group. Furthermore, the treatment with different doses of PREOG alone displayed no notable immunoreactivity to the tested apoptotic marker (Figure 4(a)).

Figure 4(b) indicated that in the cadmium group, COX-2 cascade was potentiated as presented by the amplified intensity of the immunostaining of COX-2 in the seminiferous tubular cell cytoplasm when in contrast to control. The different doses of PREOG mitigated the cadmium effects and decreased significantly the positive stained COX-2 seminiferous tubular cells cytoplasm when in contrast to the cadmium group. Additionally, immunoreactivity displayed for the tested COX-2 was insignificant in the treatment with different doses of PREOG alone oral administration (Figure 4(b)).

In Figure 4(c), the immunohistochemical analysis displayed that in the cadmium group and control group, TNF-α cascade was encouraged as seen in the enhanced immunostaining signal for TNF-α. Cotreatment with different doses of PREOG rescinds the cadmium effects and decreases the amount of positively stained TNF-α immunoreactivity seminiferous tubular cells cytoplasm, compared to the cadmium group. Also, the displayed immunoreactivity for TNF-α was insignificant when treated with different doses of PREOG alone treated groups (Figure 4(c)).

### 3.9. The Histopathology Post treatment With PREOG/Cadmium

**Figure 5:**
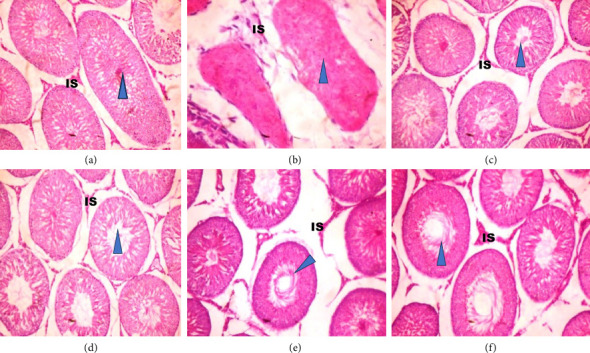
The photomicrograph of the testicular tissues in adult Wistar rats treated with cadmium and PREOG. (a) (control), (b) (3 mk/kg CdCL), (c) (100 mg/kg PREOG), (d) (200 mg/kg PREOG), (e) (100 mg/kg PREOG + cadmium), and (f) (200 mg/kg PREOG +cadmium). Slides were stained with H/E (×100).

Figure 5 shows the histopathology of the testes of adult Wistar rats exposed to PREOG and cadmium. In this figure, the control and different doses of PREOG normal testicular architecture were observed At the same time, in the cadmium-treated group (B), necrotic spermatogenic epithelial cells, the testes are smaller than normal and a bit firm in consistency, as shown by the blue triangle. Some spermatogenic epithelial cells in cadmium + PREOG groups were altered as designated by the black arrows; aside from that, normal testicular architecture is maintained. The capital letter black ISshape is in the interstitial space (Figure 5).

In the histological evaluation of the testes of the control, 100 and 200 mg/kg PREOG treatment groups (A, C, and D) indicated normal morphology of matured active seminiferous tubules (blue triangle) with spermatogenic cells at different stages of development, and the lumen is impacted with sperm cells and the interstitial space (IS) is normal lined with Leydig cells. The CdCL2 treated group (B) indicated a loss of testicular architecture of the seminiferous tubules with an impacted fibrous lumen without any sperm cells and distorted interstitial space. The altercation of the testicular architecture by CdCL2 was abrogated by the different doses of PREOG. Figure 5 (Groups E and F) indicates normal testicular structure and the different spermatogenic cells.

## 4. Discussion

Cadmium is a common environmental intoxicant with an extensive distribution globally. Both man and animals are predisposed to this intoxicant through drinking water, food, cigarette smoke, and agricultural and industrial products [66, 67]. Several scientific studies on cadmium-induced testicular pathogenesis or infertility show that of the numerous pathways of cadmium intoxication, oxidation-mediated toxicity represents the major route [15, 68], and others, such as the production of pro-inflammatory cytokines like TNF-α, incite rapid inflammatory reaction and enhance testicular germ cell apoptotic process [69, 70]. Recently, male infertility mediated by oxidative stress and inflammation has been managed successfully using nutritional supplementation such as vitamins, amino acids, minerals, and phytonutrients with significant antioxidant capability [71–73]. As the cascade of male infertility intensifies, it has become imperative for more safer and affordable approaches in the management, especially when environmental intoxicant like cadmium has been identified as the causative factor. With this in mind, this research was conducted to elucidate a novel awareness of the antiapoptotic, antioxidant, anti-inflammatory, steroidogenic, spermatogenic, tissue-protective, and fertility properties of PREOG against cadmium-induced reprotoxicity in adult male rats. From our result, PREOG plays a protective role by averting the reprotoxic effects of cadmium in adult male rats and enhancing reproductive performance.

Body weight gain is vital when evaluating the effects of environmental exposure to toxic substances in an animal [74, 75]. Cadmium has been reported to induce a significant decrease in body weight gain following treatment [76, 77]. In other studies, the body weight gain was unchanged for laying hens following cadmium treatment, while a slight increase was reported in cadmium-treated cocks [78]. In our study, the average body weight gains in the cadmium-treated rats increased significantly, and contrary to the significant decrease in testicular weight recorded from the same group of rats, the epididymal weight decrease was insignificant. This finding of decreased testicular weights by cadmium treatment aligns with what was documented by [76]. We also reported a significant decrease in GSI and EPI by cadmium treatment; the testicular biometry (left and right testicular length/diameter) was, however, unperturbed by the oral cadmium treatment. This is similar to what was reported by [79] who recorded reduced GSI value in adult male rats treated with yaji and cadmium. In another study, Shojaeepour et al. reported decreased GSI and left/right testicular diameter/length by cadmium treatment [80] while Lozi et al. and Mouri et al. reported unperturbed GSI and testicular biometric values following cadmium treatment [81, 82]. The unperturbed testicular biometry and epididymal weight could be associated with experimental protocol [65]. Nonetheless, cotreatment with different doses of PREOG abrogated the lethal cadmium effects on GSI/EPI values and enhanced significantly GSI/EPI values compared to the control. Oyeyemi and Ajuwon documented that increased GSI and EPI value can be associated with enhanced male reproductive potential [52].

The role of oxidative and antioxidative process in cadmium induced testicular intoxication has been reported in several studies [74, 83]. The insults of oxidative stress on the DNA and lipids of the spermatozoa are vital in the pathogenesis of male infertility [84]. The MDA value (lipid peroxidation), H_2_O_2_, and GSH are veritable indicators of oxidative stress-mediated pathologic conditions [77, 85]. In this study, oral cadmium chloride reduces testicular GSH value significantly and increases testicular production of hydrogen peroxide (H_2_O_2_) and lipid peroxidation (MDA value) significantly, while at the same time, the GPx value and the antioxidant enzymes viz; SOD and GST values were significantly reduced (Table 3). Findings in consonance with this were documented by [86–88]. However, oral administration of PREOG shielded the testes from cadmium-induced oxidative stress via downregulating MDA/H_2_O_2_ value and upregulating GSH value significantly, while at the same time, GPX values and testicular antioxidant enzymes, namely, SOD and GST values were notably upregulated. A finding on the protective role of PREOG was also documented by Alabi et al. in the kidney of Wistar rats [44].

The continuous reoccurrence of oxidative stress is associated with worsening inflammatory conditions, which is a known path that mediates malfunctioning of the testes by cadmium intoxication [89]. In this study, serum inflammatory response was evaluated for pro-inflammatory cytokines such as TNF-α and IL-6. The administration of cadmium chloride increased serum level of TNF-α significantly compared to the control group. There was an increase in serum level of IL-6, but it was insignificant. Yu et al. also reported increased testicular TNF-α by cadmium intoxication [77], and upregulation of IL-1β and TNF-α was documented by [70]. However, the administration of PEOG abrogated the cadmium effects and downregulated TNF-α significantly, and the decrease of serum IL-6 value in our study was statistically insignificant. Alabi et al. also recorded decreased plasma concentration of IL-6 and TN-α values by PREOG [44]. COX-2 is a known enzyme that enhances the production of prostanoids like prostaglandin E_2_ (PGE_2_). And PGE_2_ is indicated in a lot of diseases associated with inflammation. Thus, COX-2 is upregulated upon exposure to inflammatory cytokines like TNF-α [90–92]. In this study, oral administration of cadmium chloride for 8 weeks upregulated immunoreactivity COX-2 and TNF-α in the seminiferous tubular cell cytoplasm compared to the control group. Similar findings were documented by [93, 94]. Nonetheless, the different doses of PREOG treatment downregulate immunoreactivity COX-2 and TNF-*α* in the seminiferous tubular cell cytoplasm. Several studies have reported that the activation of NF-κB is an indispensable route for cadmium-induced inflammatory processes [77, 95]. One can therefore hypothesize the oral administration of PREOG-protected testicular cadmium-induced inflammatory damage via NF-κB/COX-2/TNF-α pathway.

An apoptotic signal pathway can be instituted by an enhanced inflammatory process or an unabated generation of ROS in the testes [96]. The germ cell apoptosis of the testes is a highly coordinated process associated with programmed cell death, and it is characterized by the formation of apoptotic bodies, membrane blebbing, DNA fragmentation, cell shrinkage, etc. [97, 98] thus regulating the spermatogenic process and male fertility potential. In this study, the oral administration of cadmium chloride disrupted the coordinated apoptotic process by enhancing the number of extricated apoptotic bodies, which was affirmed by the intense signals of executioner caspase-3. Similar findings to this were documented by [99–101]. Nevertheless, cotreatment with the various doses of PREOG suppresses the expression of caspase-3, which is indicative of the attenuating role of PREOG against the insult of cadmium on testicular germ cell apoptosis (Figure 4(a)).

The Sertoli and somatic cells that provide nutrition and support needed for germ cells proliferation are all situated in the seminiferous tubules [102]. The length of seminiferous tubules correlates positively with testicular length. Thus, any pathologic insult on the seminiferous tubules will culminate into the degeneration of Sertoli and germ cells, consequently impairing spermatogenesis [103]. Seminal deficiencies and sperm deformities are culpable for the soaring prevalence of male fertility decline in humans and animals and even with the milestone achievement by researchers in this field, and medical and veterinary practitioners still see the identification and management of spermatogenic failure as a delightful challenge [104, 105]. In this study, oral treatment with cadmium chloride reduced sperm motility, livability, and sperm count when in contrast to control, also total sperm morphological abnormality/deformity, the head, midpiece, and tail abnormality/deformity were increased in greater proportion after oral dosing the adult male rats for 8 weeks with cadmium chloride. This finding aligns with what was documented by [76, 100, 106, 107] who reported inhibited sperm motility, viability and concentration, increased deformity index, and number of dead/abnormal sperm cells following cadmium administration. However, this diminished sperm motility, livability, and sperm concentration recorded in our study were abrogated by the treatment with different doses of PREOG. Sperm motility, livability, and sperm count were even enhanced. The sperm total morphological abnormality, the head, midpiece, and tail deformities were averted also by the different doses of PREOG administered.

To ensure optimal performance of the male reproductive system in light of potential fertility threats such as cadmium exposure, it is crucial to promote androgen synthesis and stimulation to maintain normal spermatogenic processes [108]. One vital androgen that regulates important reproductive activity and function in males is the testosterone. Testosterone is synthesized by a series of processes in the Leydig cells. The mitochondria StAR protein translocates cytoplasmic cholesterol, and P450scc or CYP11A1 converts the cholesterol to pregnenolone [109]. The pregnenolone is then transported to the smooth endoplasmic reticulum, and catalytically, 3β-HSD changes pregnenolone to progesterone, with the help of CYP17AI the progesterone is changed to 17α-hydroxyprogesterone and androstenedione. Finally, catalytically 17β-HSD changes androstenedione to testosterone [110]. In this study, steroidogenic gene expression levels for the following genes: steroid acute regulatory (StAR) and 3β- and 17β-hydroxysteroid dehydrogenase (HSD) genes expression were assayed with real-time quantitative polymerase chain reaction (RT-qPCR), and our result indicated that oral cadmium chloride treatment for eight weeks downregulates the expression of StAR gene and 3β- and 17β-HSD gene expression significantly compared to the control group. This finding aligns with what was documented by [76, 77, 89, 111, 112]. The downregulated steroidogenic genes reported in this study also correlate positively to the steroid hormone biosynthesis, as the cadmium-treated adult male Wistar rat serum testosterone level was notably decreased in contrast to the control. This finding agrees with what was documented by [89, 113]. In this study, cadmium treatment decreased serum LH levels significantly in contrast to the control. However, serum FSH level was elevated significantly by the cadmium treatment. This finding corresponds with what was documented by [83, 114] who recorded that the elevated FSH/LH values and impeded testosterone synthesis by cadmium treatment are an obvious pointer of a broken hypothalamic-pituitary testicular axis or an impaired Leydig cell function. However, the oral administration of different doses of PREOG mitigated the cadmium effects on steroidogenic genes and enhanced steroidogenesis as seen in the increment of serum testosterone value.

Histological examination of the testes in adult male Wistar rats treated with 3 mg/kg cadmium chloride for 8 weeks revealed necrotic spermatogenic epithelial cells, decreased spermatogenic processes, and reduced testicular sizes than normal, with a bit of firm consistency with normal H &E histopathologic staining. This finding is similar to what was documented by [115] who reported deteriorating seminiferous tubules and the absence of spermatogenesis in cadmium-treated rats due to its degenerative effects and necrosis. Other studies with morphological lesions resulting from cadmium testicular intoxication were documented by [116–118]. However, the different doses of PREOG mitigated and restored the histopathologic effects of cadmium on the testes of treated rats.

In this study, we have been able to establish that oral administration of cadmium chloride to an adult male Wistar rat for 8 weeks was detrimental to its reproductive index as a result of the decrease in GSI and EPI value, enhanced inflammation and oxidative stress, enhanced apoptotic process, decreased in semen quality, and downregulation of steroidogenic genes which correspond to the decreased testosterone level and altercation of normal testicular histology. These findings are similar to what has been documented in several studies on the reprotoxic effects of cadmium in man and animals and the usage of different substances to partially or completely prevent this environmental intoxicant from impairing the male reproductive capacity [119]. In the same way, we have also elucidated a novel awareness of the antiapoptotic, antioxidant, anti-inflammatory, steroidogenic, spermatogenic, and tissue-attenuating role of PREOG against cadmium-induced reprotoxicity in adult male rats. To affirm if the improved reproductive parameters in this study translate to improved reproductive outcomes, a fertility test was done to assess the ability of the exposed adult male Wistar rats to cadmium for 8 weeks to sire offspring. From our result, the fertility index, parturition, and gestation index of the treated male that cohabited with untreated females decreased by 50% in contrast to the control, and there was also a significant decrease in the percentage of pups delivered (%) in the cadmium group compared to the control group. However, the mean birth weight and survival index for the first 7 days of the pups' lives were insignificant in this study (Table 5). Findings similar to this were documented by [120, 121] who reported decreased male reproductive performance, increased resorption rates in pregnant females, decreased gestation index, and number of pups delivered following exposure to sodium fluoride. We can hypothesize a reduction of libido in the treated adult male since there was a downregulation of the steroidogenic gene and a corresponding decrease in serum testosterone concentration in this study. A similar finding to this was reported by [122, 123], since testosterone is needed for effective male copulatory activities in rodents [123]. The impaired gestation/parturition index in this study could be as a result to the reduced semen quality/quantity reported earlier consequently eliciting pre-/postimplantation loss. This is similar to what was reported by [64, 122, 123]. Khouri and El-Akawi also reported that decreased reproductive capacity from poor sperm quality/quantity results in preimplantation loss [124]. From this result, the detrimental impact of cadmium on the reproductive capacity of adult male Wistar rats translates to the impaired fertility outcome as seen in. However, the treatment with different doses of PREOG mitigated cadmium effects and enhanced the reproductive performance of adult male rats (Table 5).

In conclusion, our finding has further affirmed the reprotoxic effects of cadmium in adult male Wistar rats as seen in the decline of semen quality, steroidogenesis, enhanced apoptosis, impaired reproductive outcome, and the aggravated histopathological changes of the testes. We also reported for the first time the protective role of PREOG in averting testicular apoptosis, inflammatory processes, oxidative stress, and histopathologic changes due to cadmium intoxication. We reported also that the inflammatory process was abrogated along the COX-2/TNF-α pathway. For the first time here, we documented that the protective role of PREOG correlated positively with reproductive outcomes. We can say, therefore, that the deleterious effect of cadmium on male fertility potential was abrogated by PREOG, and fertility performance enhanced. Further research is needed to explore the protective role of PREOG in the male and female offspring of pregnant dams exposed to cadmium during gestation and lactation.

## Figures and Tables

**Table 1:** Properties of primers used in this study.

Genes/sequence	Genes	Sequence
1	STARF	CAGCAGGAGAATGGAGATGAA
2	StAR	GGTCCACCAGTTCTTCATAGAG
3	BHSDF	TAGTGCTGCCTGAGACTACT
4	BHSDR	CTGCAGCTCTTTCTACAACCT
5	B17HSDF	CACTCTTTGCCCACTATCA
6	B17HSDR	TCGCATCGCAGTCAAGAA
7	FACTB	AGGCATCCTCACCCTGAAG
8	RACTB	CACACGCAGCTCATTGTAGA

*Note:* StAR; steroidogenic acute regulatory proteins, βHSD; beta hydroxysteroid dehydrogenase, 17β-HSD; 17 beta hydroxysteroid dehydrogenase, rat ACTIN (ACTB); Beta actin, R; reverse transcriptase, F; forward transcriptase.

**Table 2:** Testicular biometry of adult Wistar rats exposed to Cd and PREOG.

Testicular biometry/group	Average testicular weight (g)	Epididymal weight (g)	Weight difference (g)	Left testes length (mm)	Right testes length (mm)	Left testes diameter (mm)	Right testes diameter (mm)	GSI (%)	EMI (%)
Control	1.34 ± 0.11	0.56 ± 0.33	53.01 ± 0.30	16.90 ± 3.31	16.87 ± 1.31	7.44 ± 1.52	7.02 ± 1.47	2.52 ± 0.2	1.06 ± 0.1
Cd	1.01 ± 0.19^a^	0.47 ± 0.24	50.89 ± 0.39^a^	16.98 ± 0.76	17.00 ± 0.50	8.37 ± 1.05	7.86 ± 1.55	1.98 ± 0.1^a^	0.92 ± 0.1^a^
100 mg/kg Ext	1.35 ± 0.15^b^	0.72 ± 0.15^b^	53.48 ± 0.02^ab^	16.10 ± 1.61	16.85 ± 0.98	6.32 ± 0.10	6.94 ± 2.25	2.52 ± 0.3^b^	1.35 ± 0.2^ab^
200 mg/kg Ext	1.37 ± 0.11^b^	0.72 ± 0.08^b^	54.88 ± 0.32^ab^	17.82 ± 1.57	17.74 ± 0.64	7.00 ± 1.61	7.12 ± 1.01	2.49 ± 0.2^b^	1.31 ± 0.2^ab^
100 mg/kg Ext + Cd	1.29 ± 0.43	0.48 ± 0.12	51.63 ± 0.58^ab^	17.68 ± 1.72	18.24 ± 1.19	7.33 ± 1.41	6.13 ± 0.52	2.49 ± 0.2^b^	0.93 ± 0.1^a^
200 mg/kg Ext + Cd	1.20 ± 0.08^ab^	0.62 ± 0.15	52.13 ± 0.19^ab^	15.69 ± 2.02	16.23 ± 2.70	5.72 ± 1.79	8.04 ± 4.18	2.30 ± 0.2^b^	1.19 ± 0.1^ab^
*P*	0.1072	0.1982	0.0001	0.4952	0.3041	0.0778	0.7693	0.0020	0.0001

*Note:* Each value expressed as mean ± SD. Using one-way ANOVA (*p* < 0.05) indicates a notable difference across all groups. Cd, CdCL_2_; Ext, PREOG.

Abbreviations: EMI = epididymal index, GSI = gonadosomatic index.

^a,b^Superscripts (a & b) indicate notable difference from control and Cd groups.

**Table 3:** Semen quality of adult Wistar rats treated with Cd and PREOG.

Semen parameters/groups	Sperm motility (%)	Sperm viability (%)	Sperm count (10^6^/sperm/mL)	Head defect (%)	Mid-piece defect (%)	Tail defect (%)	Total morphological defect (%)
Control	96.80 ± 1.30	83.40 ± 3.21	84.00 ± 7.62	0.26 ± 0.39	5.89 ± 0.85	4.13 ± 0.90	10.08 ± 0.82
Cd	56.40 ± 7.83^a^	54.60 ± 5.32^a^	72.60 ± 4.67^a^	1.79 ± 0.73^a^	11.96 ± 1.94^a^	14.83 ± 2.52^a^	28.58 ± 4.77^a^
100 mg/kg Ext	94.20 ± 2.59^ab^	91.20 ± 3.90^ab^	97.80 ± 5.31^ab^	0.66 ± 0.42^b^	5.36 ± 0.63^b^	7.34 ± 2.44^ab^	13.37 ± 2.71^ab^
200 mg/kg Ext	90.80 ± 5.54^ab^	87.80 ± 2.28^ab^	100.00 ± 12.41^ab^	0.53 ± 0.70^b^	6.03 ± 0.68^b^	6.33 ± 1.56^ab^	12.89 ± 1.28^ab^
100 mg/kg Ext + Cd	70.20 ± 7.76^ab^	67.40 ± 3.71^ab^	103.80 ± 7.98^ab^	0.10 ± 0.22^b^	7.13 ± 1.01^ab^	5.54 ± 0.59^ab^	12.78 ± 0.64^ab^
200 mg/kg Ext + Cd	72.40 ± 7.92^ab^	71.00 ± 7.31^ab^	105.60 ± 14.08^ab^	0.43 ± 0.42^b^	6.16 ± 1.86^b^	5.69 ± 1.49^ab^	12.27 ± 3.62^b^
*p*	0.0001	0.0001	0.0001	0.0004	0.0001	0.0001	0.0001

*Note:* Each value expressed as mean ± SD. Using one way ANOVA (*p* < 0.05) indicates a notable difference across all groups. Cd, CdCL_2_; Ext, PREOG.

^a,b^Superscripts (a & b) indicate notable difference from control and Cd groups.

**Table 4:** Impact of oral PREOG leaves/cadmium on the oxidative status.

Groups	H_2_O_2_ (nmol/g protein)	GSH (mg/g protein)	MDA (nmol/g protein)	GPx (U/mg protein)	GST (U/mg protein)	SOD (U/mg protein)
Control	35.54 ± 1.79	137.00 ± 11.73	4.88 ± 3.19	72.95 ± 9.16	26.43 ± 4.67	66.89 ± 6.42
Cd	48.87 ± 1.85^a^	107.01 ± 12.04^a^	13.09 ± 4.17^a^	49.09 ± 8.92^a^	14.56 ± 5.01^a^	40.78 ± 7.34^a^
100 mg/kg Ext	34.68 ± 0.66^b^	131.75 ± 1.42^b^	4.09 ± 6.19^b^	77.97 ± 8.94^b^	23.46 ± 3.20^b^	66.89 ± 6.55^b^
200 mg/kg Ext	34.54 ± 1.09^b^	137.00 ± 11.73^b^	4.88 ± 3.19^b^	72.95 ± 9.16^b^	26.43 ± 4.67^b^	66.89 ± 6.42^b^
100 mg/kg Ext + Cd	42.03 ± 0.14^ab^	119.09 ± 11.06^a^	8.09 ± 3.93^b^	62.56 ± 6.56^ab^	19.01 ± 2.07^a^	53.72 ± 3.87^ab^
200 mg/kg Ext + Cd	40.45 ± 1.01^ab^	118.04 ± 11.58^a^	7.97 ± 4.65	65.98 ± 7.08^b^	18.98 ± 9.82	52.07 ± 8.98^ab^
*P*	0.0001	0.0006	0.0306	0.0003	0.0291	0.0001

*Note:* Each value expressed as mean ± SD. Using one-way ANOVA (*p* < 0.05) indicates a notable difference across all groups. Cd, CdCL_2_; Ext, PREOG; GPx; glutathione peroxidase; H_2_O_2_, hydrogen peroxide; GSH, reduced glutathione; MDA, malondialdehyde.

Abbreviations: GST = glutathione-S-transferase, SOD = superoxide dismutase.

^a,b^Superscripts (a & b) indicate notable difference from control and Cd groups.

**Table 5:** The fertility index of adult male rats exposed to Cd and PREOG.

Fertility index/birth weight/groups	Male fertility index (%)	Female fertility index (%)	Parturition index (%)	Gestation index (%)	Mean ± SD of delivered pups (%)	Mean ± SD birth weight delivered (g)	Survival index from PND1 to PND7 (%)
Control	100	100	100	100	8.8 ± 0.8	6.07 ± 0.27	90.00 ± 10.00
Cd	50	50	50	50	5.6 ± 0.6^a^	5.12 ± 2.11	82.00 ± 8.37
100 mg/kg Ext	100	100	100	100	8.2 ± 0.5^b^	6.38 ± 3.08	88.00 ± 8.37
200 mg/kg Ext	100	100	100	100	8.8 ± 0.8^b^	6.25 ± 2.30	94.00 ± 8.95^b^
100 mg/kg Ext + Cd	80	80	80	80	5.6 ± 1.5^a^	5.62 ± 3.60	88.00 ± 8.37
200 mg/kg Ext + Cd	80	80	80	80	7.0 ± 0.7^ab^	5.98 ± 2.91	82.00 ± 8.37
*P*					0.0001	0.9753	0.2511

*Note:* Each value expressed as mean ± SD. Using one-way ANOVA (*p* < 0.05) indicates a notable difference across all groups. Cd, CdCL_2_; Ext, PREOG; PND, postnatal day.

^a,b^Superscripts (a & b) indicate notable difference from control and Cd groups.

## Data Availability

The data used to support the findings of this study can be made available by the corresponding author upon reasonable request.
